# Quinolone Safety and Efficacy More Important than Potency

**DOI:** 10.3201/eid1001.030307

**Published:** 2004-01

**Authors:** Richard Frothingham

**Affiliations:** * Veterans Affairs Medical Center and Duke University Medical Center, Durham, North Carolina, USA

**Keywords:** Fluoroquinolone, drug therapy, levofloxacin, moxifloxacin

**To the Editor:** In a recent article, Scheld defines two principles for appropriate quinolone use based on the goal of maintaining class efficacy, namely avoiding unnecessary antimicrobial drug therapy and “using the agents with optimal activity against the expected pathogens” (*1*). He presents a large body of evidence supporting an inverse correlation between quinolone activity and the selection of antimicrobial drug resistance. On the basis of this concept, Scheld favors ciprofloxacin for known or suspected *Pseudomonas aeruginosa* infection and moxifloxacin for infections in which *Streptococcus pneumoniae* is likely, including community-acquired pneumonia (CAP). Preventing the emergence of antimicrobial drug resistance is certainly an important goal in drug therapy decision-making. However, this goal should be balanced by the clinical criteria of safety and efficacy.

Serious adverse drug effects in patients led to the withdrawal or restriction of four quinolones in the past decade (temafloxacin, grepafloxacin, trovafloxacin, and sparfloxacin). Safety may differ substantially among the quinolones discussed in Scheld’s review (ciprofloxacin, levofloxacin, moxifloxacin, and gatifloxacin). On the basis of spontaneous reports to the U.S. Food and Drug Administration (FDA), gatifloxacin is associated with a higher rate of torsades de pointes than ciprofloxacin or levofloxacin (p = 0.001) (*2*). Torsades cases have been reported in association with moxifloxacin, but their rate cannot be estimated with any precision by using FDA spontaneous reporting data because of the relatively small number of U.S. prescriptions (*2*). In a crossover study, a single oral dose of moxifloxacin 800 mg was associated with greater QT interval prolongation (16–18 milliseconds) than ciprofloxacin 1,500 mg (2–5 milliseconds) or levofloxacin 1,000 mg (4–5 milliseconds) (*3*). Gatifloxacin has been associated with alterations in glucose metabolism, both in prospective trials and in postmarketing surveillance. Gatifloxacin underwent two “safety-related drug labeling changes” in 2001 and is the only quinolone that carries a “warning” about disturbances in glucose metabolism. Gemifloxacin was approved after Scheld’s review and has pharmacodynamic potency similar to moxifloxacin against *S. pneumoniae*. Gemifloxacin is associated with a high rate of rashes, especially in women <40 years of age.

Serious but uncommon adverse side effects may not be recognized until drugs are used in large populations (*4*). More than 100 million prescriptions were written for terfenadine and astemizole before they were withdrawn from the market because of torsades and sudden death. On the basis of the number of U.S. prescriptions in the past decade (January 1993–December 2002), patient experience with ciprofloxacin (119 million prescriptions) and levofloxacin (44 million) is larger than with gatifloxacin (8 million) or moxifloxacin (5 million) (*5*–*7*).

The clinical efficacy of ciprofloxacin and levofloxacin is better established for a broad range of indications in comparison to the newer agents. A full discussion of the literature is beyond the scope of this letter. A simple MEDLINE (U.S. National Library of Medicine, Bethesda, MD) search in April 2003 provided the following raw numbers of peer-reviewed, randomized, controlled trials reporting clinical outcomes: >200 trials using ciprofloxacin, 28 using levofloxacin, 13 using moxifloxacin, 7 using gatifloxacin, and 6 using gemifloxacin (search terms, inclusion criteria, and exclusion criteria available from the author). The quality of these studies is quite variable, and quality is certainly more important than quantity. Most trials of the newer agents were designed and funded by industry. In general, ciprofloxacin and levofloxacin have been studied in patient populations with more severe illnesses, including nosocomial infections, than the newer quinolones. With the exception of a single moxifloxacin trial (*8*), the trials of the newer quinolones have enrolled patients with predominantly mild or moderate community-acquired infections and low overall mortality rates.

Scheld provides a table that lists case reports of clinical failures of levofloxacin for the treatment of pneumococcal infections. Some cases were associated with primary or secondary levofloxacin resistance. These case reports should not be surprising, since CAP trials regularly identify clinical failures regardless of the therapy chosen. The rate of clinical failure is best determined by data from prospective trials rather than case reports. Both levofloxacin and moxifloxacin have performed well in patients with severe pneumococcal infections, on the basis of the rates of therapeutic success and death (*8*–*10*).

Scheld’s choice of ciprofloxacin as a component of combination therapy for suspected *P. aeruginosa* infections can be affirmed. Ciprofloxacin has pharmacodynamic potency against *P. aeruginosa*, a track record of safety in large populations, and a large published literature. Ciprofloxacin has demonstrated efficacy in patient populations with severe illnesses, including nosocomial infections.

Antimicrobial drug therapy decision-making for patients with CAP and other respiratory tract infections is much more complex. Individual patient factors should be considered, including the severity of illness, coexisting illnesses, risk factors for drug-resistant *S. pneumoniae*, and risk factors for specific adverse effects. A respiratory quinolone will be an appropriate choice for some patients with CAP. Among the respiratory quinolones, a wholesale switch from levofloxacin to moxifloxacin, on the basis of pneumococcal potency alone, would be premature. Clinicians should use newer quinolones cautiously until their safety has been established in large patient populations.
